# Signaling during Kidney Development

**DOI:** 10.3390/cells4020112

**Published:** 2015-04-10

**Authors:** Mirja Krause, Aleksandra Rak-Raszewska, Ilkka Pietilä, Susan E. Quaggin, Seppo Vainio

**Affiliations:** 1 Faculty of Biochemistry and Molecular Medicine, Biocenter Oulu, Oulu University, 90014 Oulu, Finland; E-Mails: mirja.krause@oulu.fi (M.K.); aleksandra.rak-raszewska@oulu.fi (A.R.R.); ilkka.pietila@oulu.fi (I.P.); 2 Northwestern University, Feinberg School of Medicine, Chicago, IL 60611, USA; E-Mail: quaggin@northwestern.edu

**Keywords:** kidney development, organogenesis, cell signaling, mesenchymal-to-epithelial transition (MET), nephrogenesis

## Abstract

The kidney plays an essential role during excretion of metabolic waste products, maintenance of key homeostasis components such as ion concentrations and hormone levels. It influences the blood pressure, composition and volume. The kidney tubule system is composed of two distinct cell populations: the nephrons forming the filtering units and the collecting duct system derived from the ureteric bud. Nephrons are composed of glomeruli that filter the blood to the Bowman’s capsule and tubular structures that reabsorb and concentrate primary urine. The collecting duct is a Wolffian duct-derived epithelial tube that concentrates and collects urine and transfers it via the renal pelvis into the bladder. The mammalian kidney function depends on the coordinated development of specific cell types within a precise architectural framework. Due to the availability of modern analysis techniques, the kidney has become a model organ defining the paradigm to study organogenesis. As kidney diseases are a problem worldwide, the understanding of mammalian kidney cells is of crucial importance to develop diagnostic tools and novel therapies. This review focuses on how the pattern of renal development is generated, how the inductive signals are regulated and what are their effects on proliferation, differentiation and morphogenesis.

## 1. Introduction

**Figure 1:**
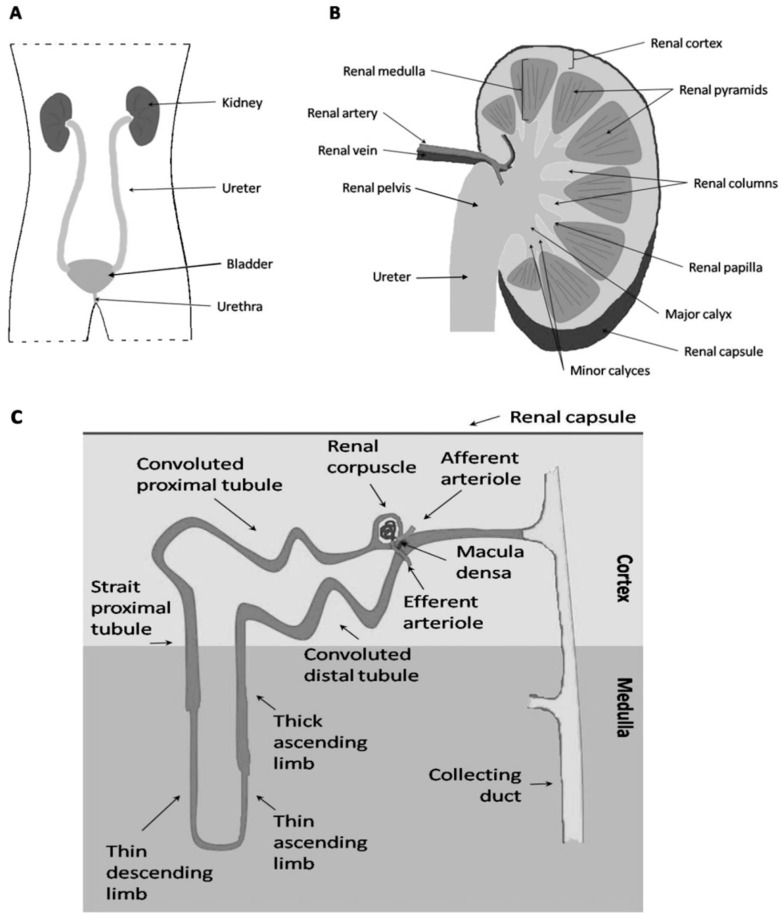
Structure and organization of the kidney. (**A**) The location of the kidney in the body; (**B**) Schematic cross section of the kidney to demonstrate the overall structure; (**C**) A closer look gives an insight on the nephron structure and orientation within the kidney with a clear distinction between the cortex and the medulla.

The kidney coordinates homeostasis in many ways. It regulates the effective blood volume and blood pressure via the renin-angiotensin system, and the excretion and uptake of plasma components, and the pH-balance of the body [1]. An overview of the kidney location in the body, the structure of the adult kidney can be seen in Figure 1. The key functional unit of the kidney is the nephron which develops from the resident progenitor/stem cells by a Wnt-signaling process as shown by our lab and others in the past [1,2,3,4].

**Figure 2:**
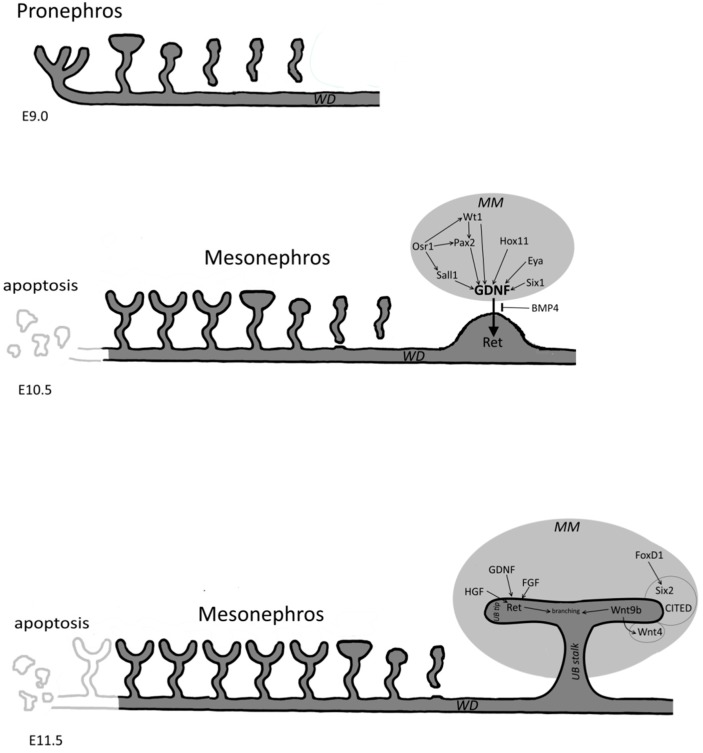
Schematic overview of the three stages of kidney development; the pro-, meso and metanephros between E9.0 to E11.5. The wolflian duct (WD) is shown in dark grey. Important signals during nephrogenesis which induce ureteric bud (UB) branching and metanephric mesenchyme (MM) differentiation are shown.

During mammalian kidney development one can identify relics of evolution that represents kidneys in more primitive life forms. Before the adult kidney (the metanephros) develops, embryos have two transient kidneys (the pro- and mesonephros) in a spatial and temporal sequence [5]. The early two forms degenerate or become part of the reproductive system while the metanephros forms the permanent secretory organ (Figure 2). Kidney organogenesis is regulated by sequential and reciprocal cell and tissue interactions. These mutual interactions mostly occur between two progenitor tissues: the epithelial Wolffian duct (WD) -derived ureteric bud (UB) and the metanephric mesenchyme (MM). Both tissue types are derivatives of the intermediate mesoderm (IM), which is a narrow cell population located between the paraxial and lateral plate mesoderm and specializes during gastrulation [6]. Kidney vasculature is also thought to originate from the IM. Although the angioblasts and the endothelial cells that migrate to the kidney organ primordium at the start of organogenesis also play a crucial role during this process [7]. The initiation of the kidney development takes place when the epithelial WD generates the ureteric bud at its extreme caudal end. Subsequently, the UB invades into the predetermined kidney mesenchyme, starts to branch and induces nephrogenesis. This all happens in a sequential manner in the metanephric blastema. Through mesenchymal epithelial transition (MET) and simple morphogenesis steps, it finally forms the nephrons. The migration of the endothelial cells into developing glomerulus is essential to create a functional nephron [5].

Currently, the only available treatment which is efficient for severe kidney diseases (e.g., end stage renal disease, ESRD) is transplantation from an immunologically suitable donor. As the number of donors is limited there is great pressure to develop novel therapies such as organ replacement transplantation with kidneys from reprogrammed, patient-derived cells. To do so, it is of great importance to understand the cell biology and detailed programming mechanisms during nephrogenesis. The latter will be covered by this review.

## 2. Review

### 2.1. Kidney Development (Pro-, Meso- and Metanephrons)

A broad range of animals develop kidneys. Between different species such as fish, frogs and mice common elements during renal development can be identified. Primitive kidney development is initiated by the expression of *Pax-2* and *Sim-1* in the IM, which in return is induced by *BMP4* secreted from the surface ectoderm [6]. These signals lead to the formation of the WD from the anterior and dorsal cells of the IM. The specialized mesenchymal cells epithelialize and start to form the nephric duct. At the same time the mesenchymal cells at the ventral IM construct the “nephrogenic cord” (NC) [6,8] which extends parallel to the nephric duct and can be identified by key transcription factors such as *Osr1*, *WT1*, *Hoxa11*, *Hoxc11*, *Hoxd11*, *Sall1*, *Six1* and *Eya1*. An overview of important signals during nephrogenesis can be seen in Table 1. This process results in the formation of the pronephros which in fish and amphibians represents the permanent kidney. It remains functional throughout adulthood. However, in mammals, the mesonephric tubules are only transient filtration units which degenerate and are replaced by the adult, or metanephric, kidney. The conservation of genetic mechanisms specifying early kidney development makes frogs (e.g., Xenopus tadpole) and fish (e.g., zebrafish larvae) excellent model systems for genetic analysis and tissue transplantation experiments [9]. The development of a second kidney is induced by the caudal elongation of the WD resulting in the formation of the mesonephros (MN). In aquatic vertebrate the MN serves as the main excretory organ, while in reptiles, birds and mammals it only represents a transient organ rudiment. Despite being transient during kidney formation in mammals, the MN is crucial for the development of other urogenital system derived organs such as gonads, adrenal gland, and also to hematopoiesis [10]. In a final step, the WD outgrows dorsally to form the UB. Signals from the UB subsequently induce nephron formation—nephrogenesis, in the adjacent predetermined MM.

**Table 1:** Genes important for nephrogenesis.

Gene Abbreviation	Full Name	Role in Kidney Development	Expression Location	References
**Growth Factors**				
**BMP4**	Bone Morphogenetic Protein 4	prevents ectopic ureteric bud outgrowth and extra ureteric bud divisions	mesenchymal cells surrounding WD and stromal mesenchyme surrounding UB stalks	[11]
**BMP7**	Bone Morphogenetic Protein 7	survival of MM	MM	[12]
**Fgf8**	Fibroblast Growth Factor 8	transition of the induced CM into RVs	CM?	[13]
**GDNF**	Glial-cell derived neurotrophic factor	induces UB outgrowth from WD, interacts with Ret	MM	[14,15]
**VEGF**	Vascular endothelial growth factor	promotes endothelial cell proliferation, differentiation	s-shaped body	[16]
**Wnt4**	Wingless-Type MMTV Integration Site Family, Member 4	mesenchymal-to-epithelial transition (MET)	cap MM, pre-tubular aggregate, nephron progenitors	[17,18]
**Wnt5a**	Wingless-Type MMTV Integration Site Family, Member 5a	nephrogenesis induction, ectopic bud formation	UB, MM	[19,20,21]
**Wnt9b**	Wingless-type MMTV integration site family, Member 9B	renewal and differentiation of nephron progenitors and normal ureteric bud branching, MET	UB stalk epithelial cells	[17,22,23]
**Growth Factors Receptors**				
**Fgfr1, Fgfr2**	Fibroblast Growth Factor Receptor 1, 2	early metanephric development	UB, MM	[24,25,26]
**Gfrα1**	GDNF Family Receptor Alpha 1	outgrowth of cells from the WD towards the MM	UB, MM	[10]
**Notch 2**	Neurogenic locus notch homolog protein 2	maturation of proximal end of the nephron	MM	[27,28]
**Ret**	Receptor tyrosine-protein kinase	initial ureteric bud outgrowth from Wolfian duct, interacts with GDNF	ureteric bud epithelial cells	[29]
**Transcription Factors**				
**BRN1**	Brain-Specific Homeobox/POU Domain Protein 1	tubule formation	S-shaped body	[30]
**FoxC2**	Forkhead Box C2	first signal in podocyte commtiment	s-shaped body	[31]
**LIM1 (LHX1)**	LIM homebox 1	initial stages of patterning in the renal vesicle	PTA, c-shaped body	[32,33]
**Osr1**	Odd-Skipped Related Transcription Factor 1	giving rise to MM	intermediate mesoderm, MM	[34]
**Sall1**	Spalt-Like Transcription Factor 1	ensures high level of GDNF production	MM	[35]
**Pax2**	Paired box gene 2	Expression in the MM ensures high level of GDNF production	UB epithelial cells and condensed MM	[36,37,38]
**Wt1**	Wilms tumor 1	ensures high level of GDNF production	cap MM-high levels, stromal MM-low levels, glomerular progenitors	[39]
**Other Signals**				
**β-catenin**	cadherin-associated protein beta	nephron formation in the early stage of kidney development	several cell types	[33,40]
**Eya1**	Eyes absent homolog 1	very early kidney development	MM	[41]
**HoxA11, HoxC11, HoxD11**	homeobox protein A11, C11, D11	early kidney development	uninduced MM	[42]
**Six1**	Sine oculis-related homeobox 1	early kidney development	uninduced MM	[32,43]
**Six2**	Sine oculis-related homeobox 2	maintain nephron progenitor cells	subpopulation of cells in cap MM	[34,44]

### 2.2. Formation of the Ureteric Bud and Induction of the Metanephric Mesenchyme

The mammalian kidney can be separated into the renal cortex and the medulla (Figure 1C). The medulla consists of the collecting ducts, loops of Henle and the interstitium [45]. Primary filtration takes place in the cortex while the concentration of the primary urine takes place in the medulla [46]. In mice the development of the metanephros is considered to begin at embryonic day (E) 10.5–11 when the UB extends from the WD towards the MM in response to inductive signals mediated by growth factors [47]. The key regulators of primary UB outgrowth and UB branching are GDNF, secreted by the MM, and the Ret receptor present on the UB cells. Secreted GDNF activates a Gfrα1/Ret receptor tyrosine kinase complex in the WD. This initiates a signaling cascade that up-regulates expression of the Ret receptor, and hence triggers the outgrowth of Ret positive cells from the WD towards the GDNF signal [10]. At E10-10.5 the first visible morphological changes in the UB development show as a broad swelling of the WD at the level of the highly restricted MM. Finally, at E11, from the most caudal part of the swollen WD, a bud emerges; growing dorsally into the MM to form the UB. The UB is an epithelial tube with a continuous lumen which is surrounded by a basement membrane [48,49]. Eventually, the elongation of the UB gives rise to the differentiation into two different structures with varying patterns in gene expression and developmental fates. One is the collecting duct derived from the distal tip of the UB by branching out. The other is the ureter and pelvis derived from the trunk of the primary UB [10,29]. Malfunction or loss of any of the signals mentioned above, leads to complete failure and kidney agenesis in mouse development [50,51].

### 2.3. Formation of Medulla and Collecting Duct System

The medulla and collecting duct system develop through continuous dichotomous branching and elongation [46,52]. The UB makes up the intrarenal collecting system which comprises the collecting ducts (CD), pelvis and calyces. It also forms the ureter and the bladder trigone [53]. The patterning of the kidney is due to branching of the UB. The epithelium grows and is being remodeled, while it retains a continuous lumen throughout its length [48,54,55]. The growth of the UB tree during nephrogenesis is tightly linked to cell proliferation. After it has invaded the MM, nine cycles of branching events between E11.5 and E15.5 create about 350 to 500 tips [52,56]. The branching of the UB follows a specific pattern, the first branching being a bifurcation which is followed by trifurcation at each of the first two tips [57]. Then branching continues via bifurcation. During this process, each tip swells to form a rounded ampulla, which is then remodeled to form two or three tips that will extend to generate new branches. One can observe a rapid branching phase, with increased cell proliferation at the tips and lower at the UB trunk. Time-lapse studies with GFP labelled tip cells have shown that the tip cells are the main progenitor cell population during UB growth. They not only produce new tip cells, but also support the elongation of the trunk [29]. It has been found that tip cells remain almost undifferentiated, while trunk cells begin to express genes characteristic of the mature collecting ducts [58,59].

It should be mentioned that studies have shown that the extracellular matrix (ECM) influences ureteric branching morphogenesis. This was demonstrated by analysing the role of laminins and integrins. ECM is a combination of secreted molecules which provide biochemical and structural support to the surrounding cells [60]. It determines a microenvironment with influence on cell behavior, proliferation, differentiation, change of shape, and establishment of polarity [61]. Laminins, collagens, nidogens and others are components of the basement membrane which forms layered cell-adherent ECMs. They are part of the protein network foundation of most cells. Lack of laminin (*Lamc1*-gene) in mice can result in development failure after E5.5 [62], and there can be UB growth and branching defects [63]. Studies with integrins (α8) knock-out mice show that these mice develop defect kidneys and they do not develop ureters [64]. Mutations like this seem to influence important signals at E11.5, like the expression of GDNF [65].

Patterns of gene expression vary during the branching process. UB tips and trunks display different patterns of gene expression [56,66,67,68]. Finally, after E15.5 the branching rate slows down. Only the trunk stretches extensively, producing the collecting duct, renal medulla and papilla. The generation of new nephrons in mice continues even after birth for 4 more days, and presumably the branching stops around the same time [69].

We can conclude that the underlying cellular mechanisms that lead to UB branching is a complex process of which we still lack complete understanding (see also Figure 2).

### 2.4. Molecular Signals that Control Ureteric Bud Growth and Branching

An intricate network of signals controls UB growth and its branching. One of the first and most important discoveries in the area of kidney development was that the MM can induce UB growth and branching as well as the reciprocal capability of the UB to induce nephrogenesis [5,70]. These processes are regulated by many different stimulating or inhibitory signals. The MM cells release factors that promote growth and branching of the UB tips. The cells (nephron epithelial and stromal cells) surrounding the UB trunks promote elongation, but suppress branching [71]. Some of the factors promoting UB growth and branching are also known to control budding from the ND (e.g., GDNF, FGFs, BMPs), but others are exclusively present during one specific process and do not play any role in the initial budding [72,73,74].

#### 2.4.1. GDNF/RET-Signaling

As mentioned earlier, GDNF/RET signaling is important during primary bud formation. However it is also crucial for UB branching throughout kidney development [71,72,75,76,77]. It has been shown that the same genes that control *Ret* expression in ND do so also in the UB tips. Ret signals are activated by many pathways such as ERK MAP kinase, PI3K, and PLCγ pathways [78,79]. As shown by several studies, they all contribute to common UB branching [50,79,80]. One of the most important function of GDNF/RET signaling is the up-regulation of different UB tip target genes including *Ertv4*, *Etv5*, *Met*, *Mmp1*, *Spry* and *Wnt11* [66,81,82]. However, only *Etv4* and *5* are necessary for the UB outgrowth from the ND [82].

#### 2.4.2. FGF Signaling

Fibroblast Growth Factors (FGF) transfer signals through FGF receptors which are important for early metanephric development. Specifically of importance are the two receptors Fgfr1 and Fgfr2. These receptors are expressed in the MM and UB around E10.5 and E11.5. Fgfr2 is expressed in all parts of the WD and plays important role in WD maintainance [83]. The lack of one of the receptors does not result in kidney malformation, but the loss of both leads to kidney agenesis. A double receptor deletion leads to the absence of *Pax2*, *Six2* and *Sall1*, even though *Eya1*, *Six1* and *Wt1* are still expressed. It also results in a loss of *Gdnf* levels at E11.5 [32].

#### 2.4.3. Six1and Sall1 Signaling

*Six1* is a homeobox protein which is expressed in the uninduced MM and is essential for early kidney development. *Sall1* is one out of four of a family of multi-zinc finger transcription factors. It plays an important role in the development of the metanehpros, and is found in the MM at E10.5. The presence of *Sall1* is dependent on the presence of *Six1*. If *Six1* is absent, *Sall1* is not expressed either. Lack of *Sall1* leads to failure in UB outgrowth form WD and results in renal agenesis [32,43].

#### 2.4.4. Wnt Signaling Pathway

The secreted lipid-modified signaling Wnt-glycoproteins (Wnt) play important roles during organogenesis; also in the developing kidney. Wnts are expressed in the ureteric bud (*Wnt5a*, *-5b*, *-6, -9b* and *-11*) as well as in the metanephric mesenchyme, (*Wnt2b*, *-4* and *-5a*). Wnt signalling between these cell types is known to induce and regulate organ growth and nephrogenesis [2,22,84,85,86]. *Wnt4* is expressed in the condensing mesenchyme and the comma- and S-shaped bodies. It is essential for the epithelialization of condensed mesenchyme [4]; its expression in nephron precursor cells is important for their development into nephrons [2]. From E13.5 onwards, *Wnt7b* is expressed in the collecting duct epithelium. It plays a crucial role in cortico-medullary axis formation [46,87]. What role the interstitial mesenchyme has in the process is not yet well understood, but it is speculated to be a critical mediator of actions described above [46]. *Wnt9b* signaling is implicated as a ureteric bud-derived inducer of nephrogenesis. It is expressed in the epithelia of the UB, and is important for the development of the transient mesonephric and permanent metanephric tubules and the extension of the Müllerian duct. The first inductive response in the metanephric mesenchyme, *i.e.*, the formation of the pretubular aggregate requires *Wnt9b* [22]. *Wnt5a* is the most recently discovered member of the Wnt family which is involved in kidney development [19,20,21]. *Wnt5a* is of great importance for the development of various organs in mice; namely the skeleton [84], midbrain [88,89], mammary gland [90], uterus [91], prostate [92], intestinal elongation [93], male spermatogonial stem cells [94], and ovarian follicles [95]. Many kidney cancers and other diseases could be linked to faulty *Wnt5a* expression [96]. Robinow syndrome is very well known, and is caused by a *Wnt5a* mutation. Symptoms are short-limb dwarfism, costovertebral segmentation defects, and abnormalities of the head, face and external genitalia [97]. Thus, faulty Wnt signaling can have a severe effect on embryo development.

During late embryonic development the CDs of the renal medulla and papilla are formed by the elongation of the UB trunks. In coordination with the simultaneous elongation of the loops of Henle from nephrons in the cortex, the cortico-medullary axis of the kidney forms. Along this axis the CDs and nephrons are patterned. During development of the medulla and papilla and also during postnatal growth of the kidney and the orientation of the mitotic spindle is an important mechanism of CD elongation [23,46,55,98]. During the process cell division is controlled by *Wnt7b* and *Wnt9b* [99,100]. If lacking *Wnt7*b, it can be observed that the kidney medulla and pelvis fail to develop in mice. The CDs that normally form are abnormally short and wide [100]. *Wnt9b*, also expressed in the UB trunks, is required for normally oriented cell division during the development of late fetal and postnatal CDs. The action is mediated through a non-canonical WNT/PCP pathway involving RHO and JNK [23]. Survival signals are of great importance during the formation of the medullary and the papillary CD. These signals include *Wnt7b*, *Hgf*, *Egf*, and laminin [46,91,101,102]. If *Wnt7b* or *Egf* is not expressed in the papillary CD cells of mice, they start to die [46,103]. It should be mentioned that during this process also the ECM (laminin presence) plays an important role by the maintenance of the Wnt7b expression, and hence promoting CD cell survival [91,101,102].

### 2.5. Nephron Progenitor Cells and Nephrogenesis

**Figure 3:**
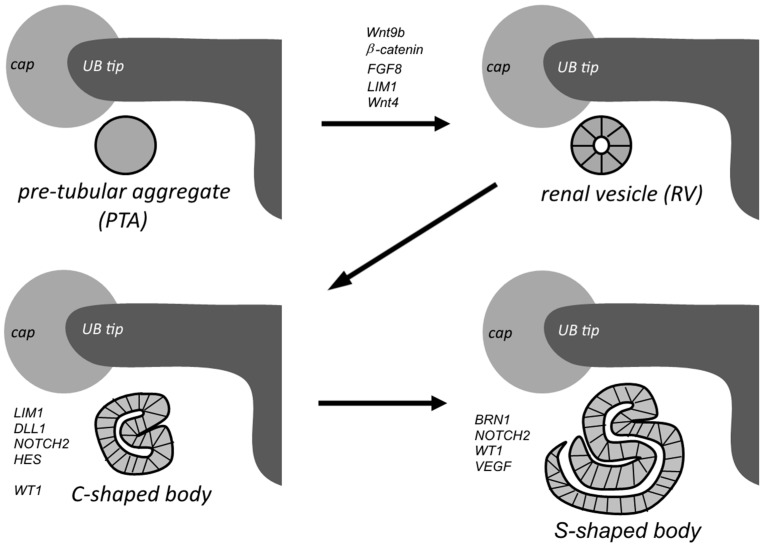
Kidney development stages. Top left: Stem/progenitor cells in the UB interact with the adjacent cap mesenchyme (CM) to form pre-tubular aggregates (PTA) just beneath the tip of the UB and differentiate into the renal vesicle (RV) (top right). Newly formed RVs undergo polarization and elongation to form Comma- (bottom left) and S-shaped (bottom right) bodies; the latter fuse with the UB tip epithelium to form the nephron.

After the primary UB budding and the subsequent invasion of the MM the UB undergoes several generations of repeated branching, as described earlier. This process is then followed by a period of reduced branching concerted with the elongation of the CD eventually leading to the formation of the collecting system. Ureter derived signals induce nephron development, initiating mesenchymal-to-epithelial transition (MET). Stem/progenitor cells within the ureteric bud epithelium interact with the adjacent cap mesenchyme (CM) [10] through GDNF and FGF signals secreted by the CM [104]. Cells begin to form pre-tubular aggregates (PTA) just beneath the tip of the UB. MM cells outside the tip area and towards the cortex remain undifferentiated. *Wnt9b* is secreted from the UB epithelium and induces CM progenitors to differentiate into epithelial renal vesicle (RV) cells [22]. Newly formed RVs undergo polarization and elongation to form Comma- and S-shape bodies; the latter fuse with the UB tip epithelium and form the nephron (Figure 3) [105]. A segmentation into specialized glomerular podocytes, the proximal convoluted tubule, loop of Henle, and the distal convoluted tubule of the nephron takes place [45]. Signaling in nephron progenitor cells and nephrogenesis will be discussed in more detail below.

#### 2.5.1. Signaling in Nephron Progenitor Cells

When the CM receives signals from the UB it can undergo one of two independent fates: (i) Self-renewal—to maintain the progenitor pool; (ii) Initiation of MET—leading to the formation of the segmented nephron [106]. The maintenance of CM progenitors ensures continued ureteric bud branching. Additionally, cellular templates for new waves of nephrogenesis are produced until the progenitor population is finally exhausted; usually, in the prenatal or early postnatal period [107]. The nephron progenitor cells of the MM express various signaling factors with essential roles during kidney development. These factors are: *Eya1*, *Cited1*, *Hox11*, *Osr1*, *Pax2*, *Sall1*, *Six1*, *Six2* and *Wt1.* The depletion of each of them in the mammalian kidney results in faulty kidney development. Deletion of genes other than *Six2* and *Sall1* leads to the loss of progenitor cells and total absence of any nephrons [8,35,39,44,92,108,109,110]. *Six2* and *Sall1* are important for the self-renewal of the progenitor population [44,106,109] and commit to the proper nephron formation at the onset of kidney development. *Six2* and *Sall1* mutations lead to early termination of the nephrogenic program, and show a severely reduced number of renal vesicles. [107]. *Sall1* is speculated to be important for the maintenance of the progenitor cells at each UB tip, ensuring that only some will undergo differentiation while the others undergo self-renewal [106].

#### 2.5.2. Nephrogenesis and the Formation of the Renal Vesicle

The start of nephrogenesis coincides with the inductive *Wnt9b* signal from the UB tip towards the CM progenitor cells [22,60]. Downstream of *Wnt9b* is another Wnt protein, Wnt4, which acts as a transcriptional determinant of distal nephron fate by the activation of the LIM homebox 1 (Lhx1) [111]. In parallel *Fgf8* is present during transition of the induced CM into RVs [4,13,74]. Genetic analysis of these two genes pointed out that *Fgf8* locates upstream of *Wnt4* [13,70,74,109,110,112]. The crucial early inductions of *Wnt9b* and *Wnt4* are caused by β-catenin phosphorylation directed by canonical Wnt signaling. Genetically elevated expression of β-catenin in the pretubular aggregate (PTA) can mimic *Lhx1* actions, however constituent canonical pathway activation blocks MET [17,113]. It is speculated that in the final phase of epithelialization *Wnt4* probably utilizes an alternative mechanism. This hypothesis is supported by the analysis of the calcium/NFAT pathway in the process [17,100,114,115,116]. After the mesenchymal cells started to aggregate, *Bmp7* is expressed in the differentiating nephron progenitor cells of the CM and also in the ureter epithelium [100,115]. The final phase of nephrogenesis in mice takes place postnataly; most of the *Six2* positive cells are lost up to 3 days of postnatal life. Currently, it is not known what causes the cessation of nephrogenesis [69].

#### 2.5.3. Nephrogenesis at Comma Shape Stage

The mature nephron develops in four stages; in stage I the epithelial RV forms a lumen and begins to ‘unwind’ to form Comma- and S-shaped bodies in stage II (Figure 3). In stage III the forming nephron vascularizes at the proximal end and finally matures to the fully functional nephron in stage IV. Which mechanism regulates the fusion of the nephrons with the ureteric tips, resulting in the generation of the luminal interconnection between the two epithelial networks is still poorly understood [105].

Immediately after the formation of the RV it polarizes in respect to gene expression in proximal to distal direction (domains) [30,44,105]. One can identify many genes critical for the specification of nephron segments. For example Notch2 signaling is crucial for the designation of proximal nephron fates (podocytes and proximal tubules) and Lhx1 regulates the patterning of distal structures [28,111]. Furthermore, Pou3f3 (Brn1) is expressed in the distal and mid-regions of the S-shaped body where its actions are essential to form loop of Henle [30]. Preceding the extension and folding of the tubular epithelium, targeted cell cycle gene expression is a distinct characteristic of the proximal-distal polarity [105]. Through a convergent expression process, *Wnt9b* (secreted from the UB) plays a role in the morphogenesis and extension of the proximal tubule [23].

The filtration unit of the nephron, called the glomerulus, develops from the proximal RV. The first gene specific to podocyte commitment in this region is Foxc2, which is expressed from the comma-shaped body stage [31]. Wilm’s tumour 1 (*Wt1*) is first expressed in the proximal nephron compartments and later on in the developing postnatal end where podocytes will form functional, highly specialized epithelium of the glomeruli [45]. Already at the S-shaped body stage, podocyte precursors and parietal epithelial cells differentiate [31]. When the parietal epithelial cells that form the Bowman’s capsule begin to express high levels of *Vegfa*, thus attracting angioblast populations from the surrounding interstitium, the glomerular capillaries are formed [117,118]

Another factor influencing the glomerular formation is *Bmp4*, found in podocytes, and also the *PdgfB/Pdgfrb* pathway which controls mesangial cell recruitment [119]. The full maturation and overall differentiation of podocytes is a complex, highly regulated process. Some factors, such as *Wt1*, are expressed throughout life and seem to function as some kind of organizer [120].

All the developmental steps described above lead to the formation of the final structures of functional kidney unit—the nephron. It consists of renal corpuscle (glomeruli), straight and convoluted tubules (proximal and distal), and loop of Henle. About 1 million of nephrons will make up the matured human kidney [45].

### 2.6. Concluding Remarks

Organogenesis is a complex process involving many iterative steps initiated and controlled by the presence or absence of very specific signals. In kidney development many various signaling pathways such as the Wnt-β-catenin pathway, the MAPK pathway, the ERK pathway and the FGF pathway play an important role in this regulation. During nephrogenesis the kidney exists of three different types (from simple to more complex) which are comprised of similar cell types and perform common renal functions. Genes involved in nephrogenesis are re-used during the sequence of the development of all three types. The development of suitable methods and protocols enabled us to study the kidney in culture [121]. This was the basis to identify important signals during nephrogenesis. A very prominent example is the Grobstein assay [122]. Grobstein and Auerbach disaggregated the MM and allowed it to re-aggregate. Early stages of development were observed after re-aggregation proving that the disaggregation of the tissue did not negatively influence induction processes. Rather recently, gene-mapping made further identification of the key renal development control genes possible. However, although we have an understanding of the basic cellular forces underlying kidney ontogenesis to be able to program various key cell types making up kidney and nephron segments, and induce differentiation in a controlled and targeted manner, further studies are necessary. Deep understanding of kidney development is the foundation for developing stem cell-based strategies to fight renal diseases. Findings from kidney organ culture have been and are paving the way for regenerative medicine. It brings us closer to the ultimate goal of engineering functional renal tissue or even a whole kidney.
